# Pulmonary Tuberculosis and Sanatorium Treatment

**Published:** 1910-06

**Authors:** 


**Pulmonary Tuberculosis and Sanatorium Treatment.** By C. Muthu, M.D. Pp. x, 201. London: Baillière, Tindall & Cox. 1909.

This survey of the scientific, the sanatorium and the social aspects of tuberculosis is the outcome of ten years' observation and work by the author in open-air sanatoria. It gives the matured convictions of one whose life has been given up to this kind of study. His experience has been large, and he is more than ever convinced that the sanatorium movement has not only revolutionised the treatment of consumption, but has shown to the world a way for the attainment of larger health and more wholesome living. He considers that open-air sanatoria are really doing the work of the State in that, as so many centres of education, they teach the people the gospel of fresh air to improve their stamina and efficiency.

As regards the question of communicability of the disease, he is quite in accord with those who maintain that tuberculosis possesses the lowest communicability of any of the infectious diseases, and that if ordinary precautions are taken for the disposal of the sputum, the risk of personal communication is very small.

Dr. Muthu holds that, as regards early diagnosis, no method is superior to the ear of the trained physician, and that physical signs should detect the presence of the disease long before any other method can possibly indicate it. He considers that the tendency of the present day is to rely too much upon experimental evidences, and that there is a danger that the physician may become too dependent on the pathologist for diagnosis, when by his clinical acumen he should form a judgment even more reliable, He does not encourage the use of tuberculin as a diagnostic or therapeutic agent ; he finds that the opsonic index is not reliable, that Calmette's reaction is by no means an infallible guide, and that the X-ray method is of little use. He looks forward to the time when the medical profession will be trained to detect by physical signs the earliest evidence of commencing tuberculous mischief, and that the sanatorium will be used not so much for curing or arresting consumption as for treating suspicious cases, and nipping the course of the disease in its early periods.

He rather strongly advocates the use of various kinds of exercises, such as breathing, singing, reading, and manual exercises, as part of the sanatorium routine ; he also finds benefit from formaldehyde inhalations, the use of static electricity, and from Kuhn's Suction Mask.

Nothing is more difficult than to appraise the value of any one vaunted method of dealing with a disease so complex as pulmonary tuberculosis. There can be no objection to many of these methods when carried on in conjunction with sanatorium life, but on the other hand the evidence before the world at the present time is that the principles of life in the sanatoria are by far the most important, and that no minor questions should be allowed to detract from them. Dr. Muthu's book gives an excellent outline of what this kind of life should be, and is worthy of careful study by all who have reason to take an interest in the subject.

We observe on pages 43 and 134 that Dr. Trudeau's name is given as Tradeau.

